# Digital skills for medical students – qualitative evaluation of the curriculum 4.0 “Medicine in the digital age”

**DOI:** 10.3205/zma001353

**Published:** 2020-11-16

**Authors:** Sebastian Kuhn, Natalie Müller, Elisa Kirchgässner, Lisa Ulzheimer, Kim Lucia Deutsch

**Affiliations:** 1 Universität Bielefeld, Medizinische Fakultät OWL, AG 4 - Digitale Medizin, Bielefeld, Germany; 2 Universitätsmedizin der Johannes Gutenberg-Universität Mainz, Zentrum für Orthopädie und Unfallchirurgie, Mainz, Germany

**Keywords:** digital transformation, curriculum 4.0, digital competencies, medical studies, Digital Supply Act

## Abstract

**Introduction: **The digital transformation has far-reaching implications for the qualification profile of medical students, which have not been addressed in medical studies so far.

**Teaching concept: **The competence-oriented blended learning curriculum “Medicine in the digital age” has been implemented at Mainz University Medical Centre since 2017. It represents a curricular reform project of the “Curriculum 4.0” program (Stifterverband). In six modules, the qualification requirements for digital skills are addressed.

**Evaluation Methodology: **The qualitative evaluation of the course concept took place in the form of semi-structured interviews. All 58 participants from five courses were interviewed.

**Results: **Using the “Qualitative Content Analysis” according to Philipp Mayring, the statements were divided into deductive main categories (process, content, methodology, learning success, learning experience and conclusion). The results reflect the student's view of the curriculum and the current qualification needs that still need to be specialised.

**Discussion: **The didactic teaching of digital skills is a relevant and highly topical component of the further development of medical studies. In this development, the focus is not only on technological skills, but also on the

## Zusammenfassung

**Einleitung: **Die digitale Transformation hat weitreichende Implikationen für das Qualifizierungsprofil von Medizinstudierenden, welche bisher im Medizinstudium nicht adressiert wurden.

**Lehrkonzept: **Das kompetenzorientierte Blended-Learning-Curriculum „Medizin im digitalen Zeitalter“ wurde seit 2017 als curriculares Reformprojekt im Rahmen des Förderprogramms „Curriculum 4.0“ des Stifterverbandes an der Universitätsmedizin Mainz implementiert. In sechs Modulen soll der Qualifizierungsbedarf an digitalen Kompetenzen adressiert werden.

**Evaluationsmethodik: **Die qualitative Evaluation des Kurskonzepts erfolgte in Form semi-strukturierter Interviews. Hierbei konnten alle 58 Teilnehmenden aus fünf Kursen befragt werden.

**Ergebnisse: **Anhand der „Qualitativen Inhaltsanalyse“ nach Philipp Mayring wurden die Aussagen in deduktiv gebildete Hauptkategorien (Ablauf, Inhalt, Methodik, Lernerfolg, Lernerlebnis und Fazit) unterteilt. Die Ergebnisse spiegeln die studentische Sichtweise auf das Curriculum und des aktuell noch zu spezifizierenden Qualifizierungsbedarfs wider.

**Diskussion: **Die didaktische Vermittlung digitaler Kompetenzen ist ein relevanter und hochaktueller Bestandteil der Weiterentwicklung des Medizinstudiums. Bei dieser Entwicklung stehen nicht nur die technologischen Kompetenzen, sondern insbesondere auch die Reflexion der fachlichen, rechtlichen und ethischen Zusammenhänge sowie der Umgang mit Veränderungsprozessen im Fokus.

## Introduction

Digital transformation refers to an ongoing change process, rooted in digital technologies, that is changing the health care system, clinics, practices, universities, professions and patients. It describes an increasing “super-convergence” of classical medicine with information technologies, which is transforming the previous health care system into a new, digital health care system [1], [2]. 

Developing medical practice through the introduction of digital health technologies is a complex process of change. In addition to investments in technologies, this requires a profound change in people's mentalities. Medical patient care is multifaceted and is provided by people with deep-rooted personal, social and institutional beliefs and practices. In order to successfully develop medical practice in particular, the digital transformation must be understood by the medical profession as a long-lasting, disruptive process of change and innovation that will fundamentally change roles and the skills and cooperation required for this [3], [4], [5].

The future is now! In 2020, doctors will have to advise patients not only on medications and surgeries, but also on digital forms of treatment and applying these in a differentiated manner. The “Digital Supply Act” set the course for this in November 2019 [6]. Both digital health applications in the form of smartphone apps and telemedical services will become increasingly relevant to practice this year. The agreed and mandatory establishment of the telematics infrastructure will lead to more intensive data provision and is accompanied by high levels of expertise for doctors.

The current generation of medical students, often referred to as “digital natives”, has an enormous educational need in this regard. Growing up with digital media and using them in the role of a “consumer” is not enough to acquire doctor-specific digital skills. This was also shown in a study conducted by the “Hochschulforum Digitalisierung” (University Forum for Digitisation), in which only 21% of the students were rated as “digital allrounders”. The majority of students gained experience primarily from a passive, consuming role and did not actively influence the digital environment [7], [8].

A look at previous curricula of medical studies, but also at the development of the Masterplan for Medical Studies 2020 and the National Competence-Based Learning Objectives Catalogue for Medicine (NKLM) did not pay much attention to digital competencies [9], [http://www.nklm.de]. This results in the necessity to design, implement and evaluate adequate curricular concepts that are future-oriented and meet these challenges. As a curricular reform project of the University Medical Centre Mainz, “Medicine in the Digital Age” was funded by the Stifterverband as a “Curriculum 4.0” project from December 2016. The aim of this funding programme is to promote the reorientation and further development of study programmes and to identify new approaches to solutions through curricular reform projects. The first implementation took place in the summer semester 2017 and has since been part of the elective program of the University Medical Center Mainz. Since the number of registrations exceeds the available capacities – in the summer semester 2019 184 registrations compared to 12 available places – participation is distributed randomly. 

The current publication presents the qualitative evaluation of the course concept based on 58 semi-structured interviews with all participants of five courses. The aim was to collect the students' perspective in order to incorporate the results into the iterative and agile development of the course concept, but also into the current national reform processes. Research was guided by the questions of the students' subjective learning success and the acceptance of the course concept.

## Teaching concept

The blended learning curriculum “Medicine in the digital age” consists of six obligatory learning modules, each of which consists of an approx. 2-hour e-learning unit and a 4-hour classroom teaching unit. The teaching concept can be described on three levels: the format level, the method level and the module level.

On the format level, the concept is divided into the teaching forms e-learning, face-to-face teaching and co-production. The different formats change in the course of time and are consecutive. The project planning is based on the recommendations and framework of the Curriculum 4.0 working group of the Hochschulforum Digitalisierung [10], [11]. In e-learning, the participants deal with topics of digital transformation with the help of an e-book in the run-up to the event and work out knowledge-based parts on digital medicine independently and on their own responsibility before the start of class. This includes review articles written by the lecturers, interviews with experts and curated external content for the six modules. In the face-to-face lessons, situations of the digital health system are dealt with in a practical way. Changing lecturers work on various topics in problem-solving oriented teaching concepts. Here, the explicit approach is to depict the digital transformation of medicine in an interdisciplinary and interactive way. Consequently, classroom instruction is conducted in small groups with the support of various medical disciplines (anaesthesia, surgery, medical informatics, medical ethics, psychology, paediatrics, psychosomatics, radiology, trauma surgery and orthopaedics). In addition, the team of lecturers is complemented by app developers, representatives of the State Data Protection Agency and patients in the sense of a transdisciplinary approach.

**Figure 1:**
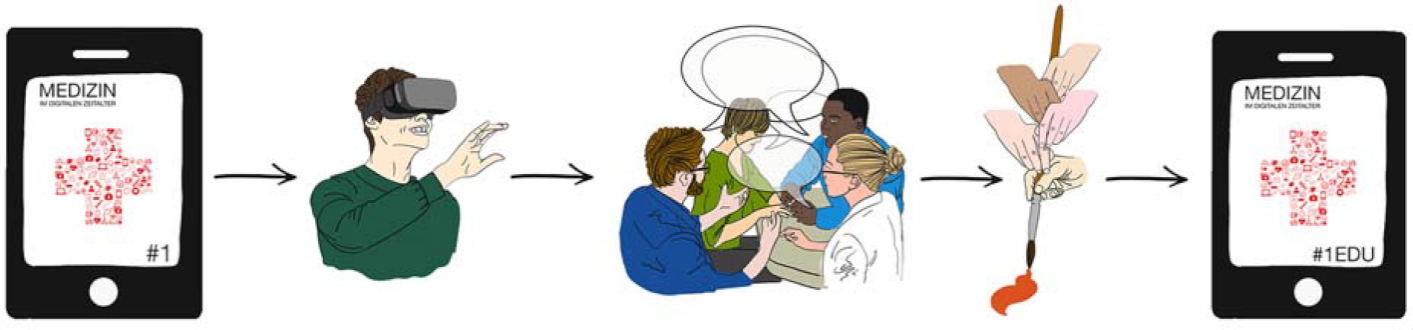
Medicine in the digital age with the elements of preparatory e-learning, face-to-face “learning by experience”, critical discussion and collaborative/co-producing of an EDU version of e-learning.

The co-productions take the form of various project work and allow for an intensive personalisation of learning, which in both analogue and digital form helps to activate the learner and to better adapt teaching to the target group. The resulting “user generated content” strengthens identification with the teaching concept and the positive learning experience. In addition, the developed content makes the learning success visible. Due to the different perspectives of the heterogeneous group of participants – students as well as teachers – diverse experiences and opinions become visible [7], [12]. The contributions will be inserted into an EDU version of the e-book after completion of the elective subject. Individual contributions will be made available for the e-learning of the following semesters (see figure 1 (Fig. 1)). 

On the method level, a distinction is made between six methods that are used in different contexts:

- working out learning goals
- keynote speeches
- workshops
- expert and patient interviews
- discussion groups
- project work

At the beginning of each module, the students collect their personal learning goals and present them to the lecturers. This allows for a comparison with the goals formulated by the instructors and enables target group-specific adjustments, especially during the workshops and discussion phase. This is followed by workshops on the module topic, which work with various methodologies. Among other things, app-based treatment concepts, video consultations and discussion groups enable students to actively and practically interact with new treatment concepts. Not only the chances and possibilities of digital medicine are considered, but also the risks and limitations of digital medicine are reflected in critical discussions of the participants with experts and lecturers. Finally, the learning goals are reviewed for their implementation and jointly reflected upon.

The module level describes the structure of the content that will be covered during the course. In total, the concept consists of six modules:

### Module 1 – digital doctor-patient communication 

**Overall learning objectives:** *The participant is able to reflect on the specific requirements of personal and electronic communication and to apply criteria for appropriate behaviour of virtual doctor-patient communication to practical examples. *

The module focuses especially on the changes in doctor-patient communication caused by digital influences. The interdisciplinary team of lecturers consists of a doctor and two psychologists. The participants are sensitized to the professional appearance in social networks and the particularities of digital communication on the three levels: doctor-patient, doctor-doctor and patient-patient. To this end, the participants will gain insights into an established online rehabilitation aftercare program, evaluate real cases of medical behaviour in social networks and discuss the advantages and disadvantages of digital communication for medical practice. In addition, digital maldevelopments, such as the uncritical use of messengers (e.g. WhatsApp^®^) in everyday clinical practice, are also addressed.

#### Module 2 – smart devices and medical apps

**Overall learning objectives: ***The participant is able to critically evaluate the benefits and risks of medical apps and smart devices and to apply them in a patient-oriented way. They will master the handling of Smart Devices and Apps in a health-specific context and will be able to reflect on the possible applications, chances and risks on the patient, physician and research level.*

The module “Smart Devices and Medical Apps” shows the digital influences on the medical consumer market. Participants will be confronted with the multitude of existing health apps and smart devices. A comprehensive examination of the qualitative assessment of an app on the levels of consumer, patient and expert will take place in the form of an independently created app review. In an expert discussion with the founders of an app-based treatment concept, the students enter into an exchange about medical, economic and political framework conditions. In a further discussion, the students talk to a doctor who has established the technology in his practice about implementation, opportunities and limitations. Smart device-based monitoring as well as the monitoring of individual vital parameters (quantified-self) are made tangible in self-experiments. 

#### Module 3 – telemedicine

**Overall learning objectives:** *After completion of the module, the participant is able to name telemedical procedures and to reflect on the chances and risks of treatment. The participant is able to apply telemedicine solutions in a patient-oriented way and to explain the necessary framework conditions of health care.*

The module “Telemedicine” uses a case study to interactively present telemedicine with the partial aspects of tele-emergency medicine, teleradiology and teleconsultation in a practical way [13]. In a video conference, the participants come into direct contact with a patient who can lead a more self-determined life thanks to several years of telemedical care.

#### Module 4 – virtual reality, augmented reality and computer-assisted surgery

**Overall learning objectives: ***The participant is able to apply and evaluate the new techniques of Virtual Reality (VR), Augmented Reality (AR) and Computer-Assisted Surgery until the end of the module.*

“Virtual Reality, Augmented Reality and Computer-Assisted Surgery”, aims at imparting the current state of development and the possible applications of VR/AR and computer-assisted surgery to the participants on the level of practical experience. Guided by experts in computer-assisted surgery, the participants work on the “Da Vinci” surgical robot simulator, use augmented reality for planning surgery and perform virtual reality laparoscopy. Here, too, the method of expert discussion is used to promote low-threshold and constructive reflection on the future role and to arouse lasting interest.

#### Module 5 – individualised medicine and big data

**Overall learning objectives:** *The participant is able to evaluate the collection and use of patient data in the area of conflict between technical and ethical principles as well as under socio-political conditions and to put it into a medical context.*

“Individualized Medicine and Big Data”, addresses the level of critical reflection of the participants. In this module, the opportunities and challenges of digitised medicine are examined and discussed from various perspectives against the background of the social framework of data protection, medical ethics and medical informatics, taking into account the contents of the entire course (access to and availability of Big Data, limitations and predictions, “right to not know”). In doing so, areas of tension in which clinically active physicians currently find themselves become visible. “What is technically possible”, “What is legally permitted”, “What is ethically justifiable” are the guiding questions. The debate takes place in particular through open discussions in the plenary with experts from the fields of medical informatics, data protection and medical ethics [14]. 

#### Module 6 – artificial intelligence 

**Overall objective:** *The participant is able to evaluate the use and benefit of artificial intelligence in medical practice in the field. Conflicts between technical and ethical principles as well as socio-political conditions are discussed and put it into a medical perspective. The participant can explain various fields of application and programs that work with artificial intelligence.*

The 6^th^ module deals with the increasing use of clinical decision support systems in everyday medical practice. The students first take a classical anamnesis and then a chatbot-assisted anamnesis (Ada Health). Individual observations of the medical and AI-assisted procedures are recorded on worksheets. In the following discussion round the advantages and disadvantages as well as the chances and risks of the application are critically reflected. In a group project, students research an AI-based clinical support system and critically reflect on the usage. The results will be presented and discussed in the plenary and will be included in the e-book in written form.

## Evaluation methodology

“Medicine in the Digital Age” has so far been conducted five times during the elective week (5 days) of the semesters 7- 9 (summer semester 2017 to summer semester 2019). Since the number of participants is limited and the number of registrations exceeded the capacities, students were randomly assigned to the course by the Department of Studies and Teaching. The team of lecturers had no influence on the selection of participants. The qualitative evaluations were performed in form of semi-structured interviews with an average duration of about 45 minutes. The interviews were conducted in a way that the research was guided by questions about the students’ subjective learning success and the acceptance of the course concept. Furthermore, the interview form proved to be a suitable instrument to obtain suggestions for the revision and adaptation of the course concept. The results of the evaluation found its way into the iterative development process. The interview guideline consists of both open and targeted questions on three sets of questions – topic-specific, event-related and reflective questions (see attachment 1 ). Deductively, six main categories (procedure, content, methodology, learning success, learning experience, conclusion) were formed. In the evaluation process, inductive subcategories also resulted. All 58 participants (36 male, 22 female) could be interviewed within 2 weeks after completion of the elective week. The total number of interviews was 25, of which 12 were individual and 13 were small group interviews. The audio recordings were transcribed, and the 1259 statements were categorized and evaluated. The evaluation process was carried out by two research assistants trained in content analysis. Any ambiguities in the data evaluation were discussed and consolidated by the project team (two other scientific assistants). The evaluation methodology was based on the “Qualitative Content Analysis” according to Philipp Mayring [15]. 

## Results

**Figure 2:**
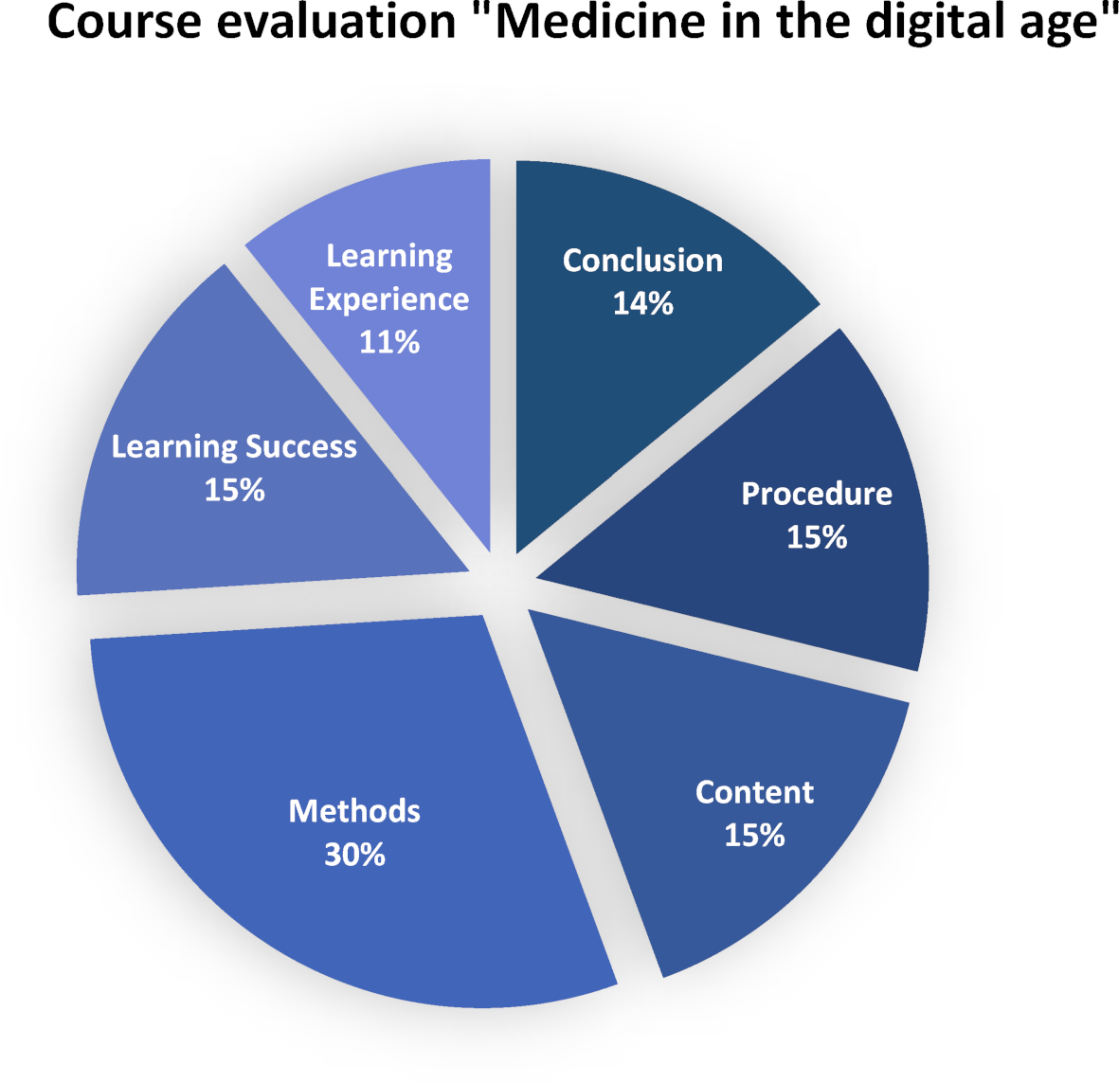
Overview of the main categories of qualitative evaluation, n=1259

The evaluation of the teaching events “Medicine in the digital age” comprises the deductively formed main categories of **procedure, content, methods, learning success, learning experience** and **conclusion** (see figure 2 (Fig. 2)). Inductively developed subcategories were assigned to the deductive main categories during the evaluation process. With the help of these subcategories, the individual main categories can now be unlocked and defined in more detail.

### Main category procedure

**Figure 3:**
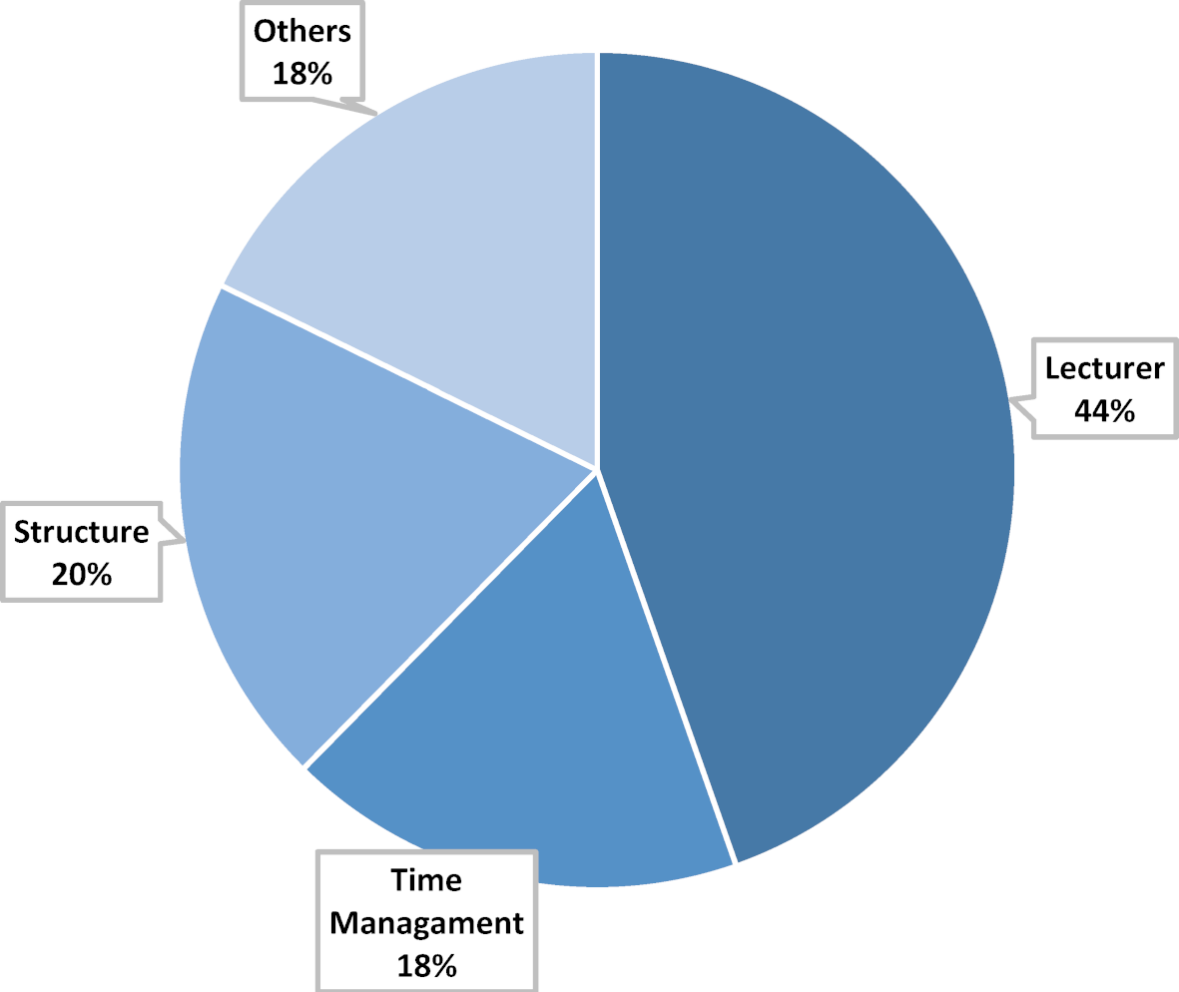
Main category Procedure, n=186

The main category Procedure (see figure 3 (Fig. 3)) includes all statements that refer to the event implementation on an organizational level. The subcategories “Lecturers”, “Structure”, and “Time Management” were created in the evaluation process. In particular, the diverse and motivated lecturers were evaluated very positively. Above all, visiting experts, such as app developers and data protection experts, made a major contribution to the course. The open discussion format was emphasized, which, according to the students, could be maintained throughout the elective week. In addition, there was consistently positive feedback on the structural organization of the event. These relate to the division of passive and active learning phases as well as the use of the different teaching formats of e-learning and classroom teaching. The students describe a smooth procedure and good organization. The temporal structure is seen positively, whereas the temporal framing is seen critically. The students’ express interest in longer teaching units.

#### Main category content 

**Figure 4:**
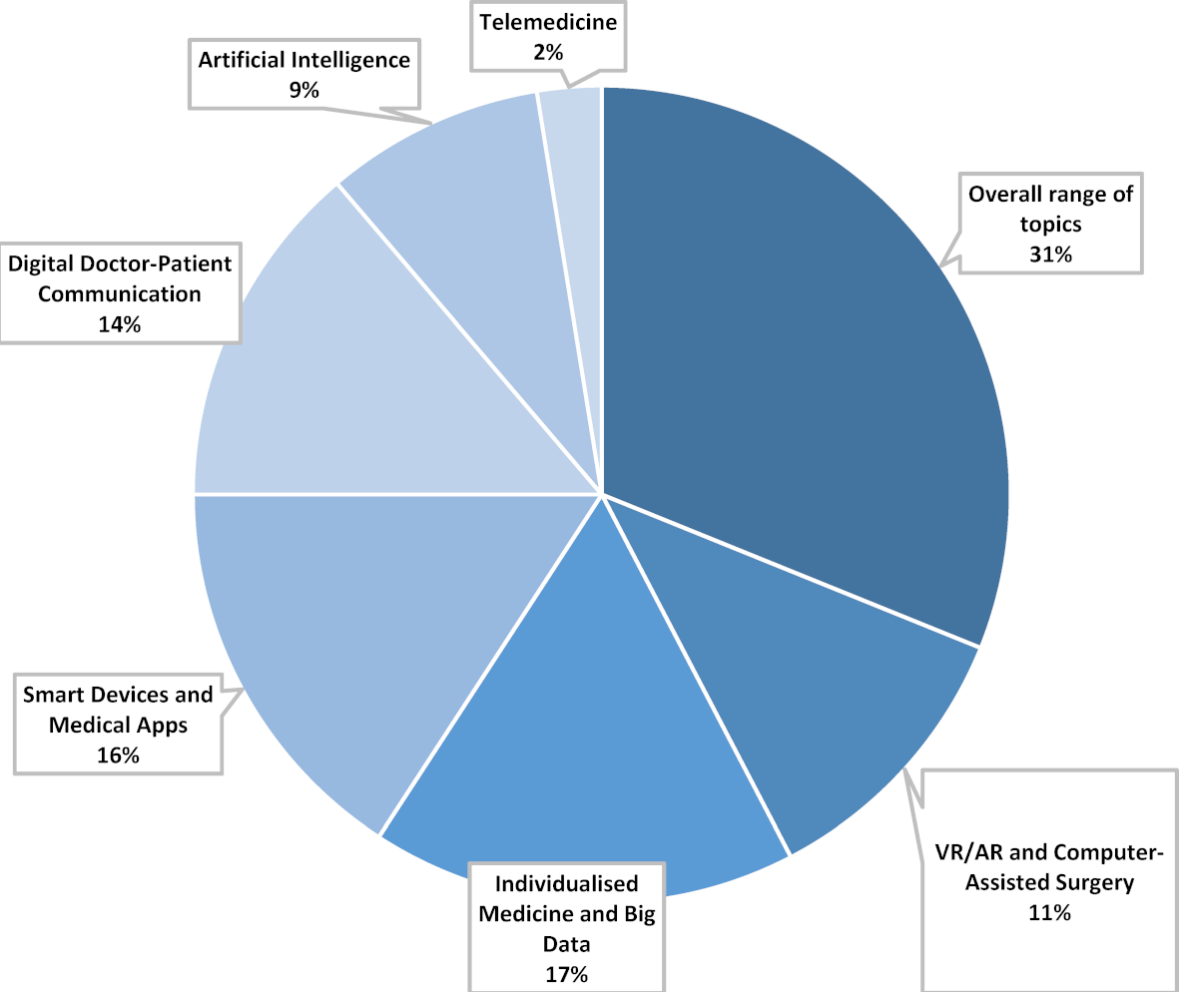
Main category Content, n=196

31% of the statements on the main category “content” refer to the variety of topics (see figure 4 (Fig. 4)). The respondents cited a general overview of the range of topics and insight into the individual practical implementations as particularly relevant. The diversity of content thus contributes to a better understanding of the scope of the topic. This includes a differentiated awareness of the current changes taking place in the medical profession. The remaining 69% of the statements can be assigned to the contents of the individual modules. In the interviews it becomes clear that the students would have wished for even more in-depth discussions on the medical-ethical and medical-legal level. Some papers have already been published on individual modules; others are in the process of being published [3], [12], [13], [14]. 

#### Main category methods

**Figure 5:**
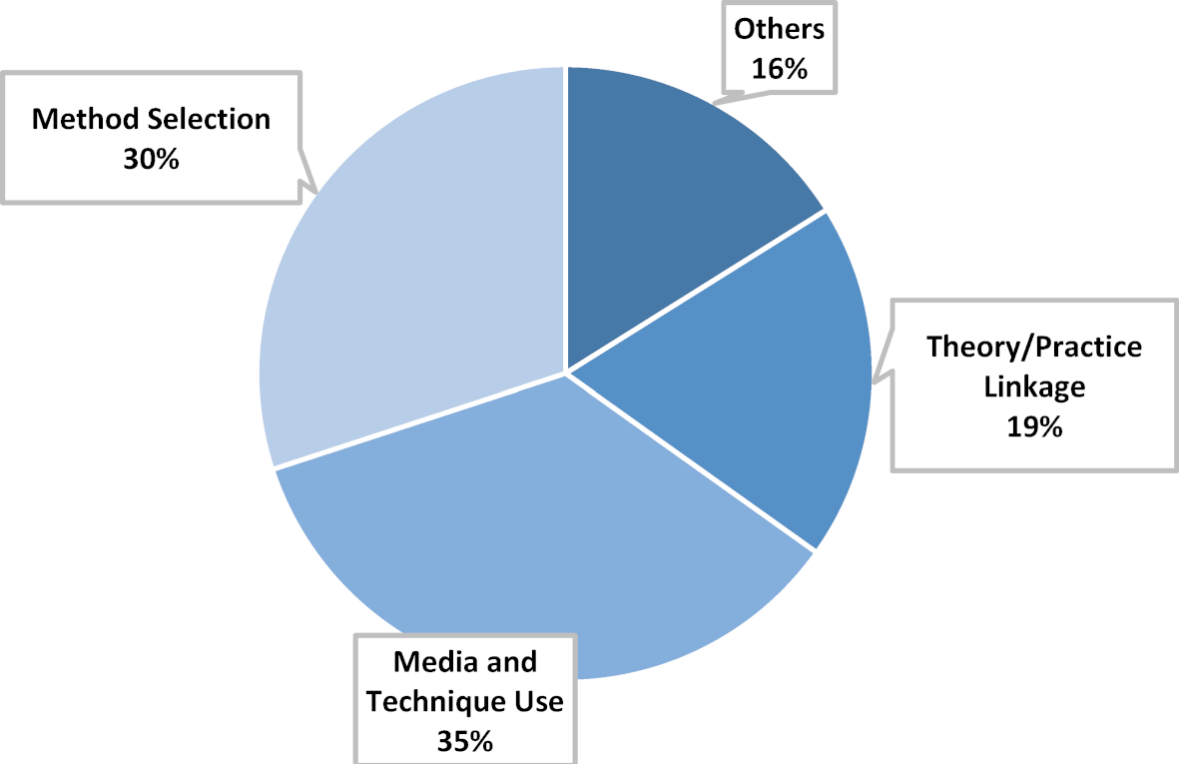
Main category Methods, n=373

The largest category with 30% of all assigned statements is the main category Methods (see figure 5 (Fig. 5)). This category includes all statements on the method application. In the evaluation process, the subcategories “Method Selection”, “Media and Technique Use” and “Theory/Practice Linkage” were formed. The students assess the selection of methods used as varied and diverse. In particular, the methods discussion and expert discussion are perceived as particularly interesting and instructive. In addition, the participants say that they appreciated the opportunity to exchange ideas with their fellow students in group work, which took the form of workshops, and to work on tasks together. The method fishbowl discussion was evaluated differently by the participants. Individual participants said that they were reluctant to discuss openly, especially at the beginning of the discussion, or on the contrary that they wanted to participate even more and express themselves more often than the method allowed. In contrast to the method selection, however, the students gave an uniform positive assessment of the theory/practice linkage. All respondents were positive about the fact that not only theoretical knowledge was learned in the course, but also practical experience was gained. With great enthusiasm, the students report about their experiences with VR/AR or the DaVinci robot simulator. However, the students recommend that the practical exercises should be given an even larger timeframe. In the statements on the use of media and technology in the course, the use of new technologies is particularly positively emphasized. Operating smart watches, tablets and other smart devices was a valuable experience for most students. In addition, the event-related e-book is evaluated as helpful for orientation before the course. The time required to read the e-book is judged to be appropriate. The use of the digital communication tool SLACK is partly praised and partly criticised, as the app was not used by all participants with the same intensity and was perceived by some students as confusing. However, the possibility of networking and information exchange, which was also possible after the course with the help of the app, were viewed positively. 

#### Main category learning success

**Figure 6:**
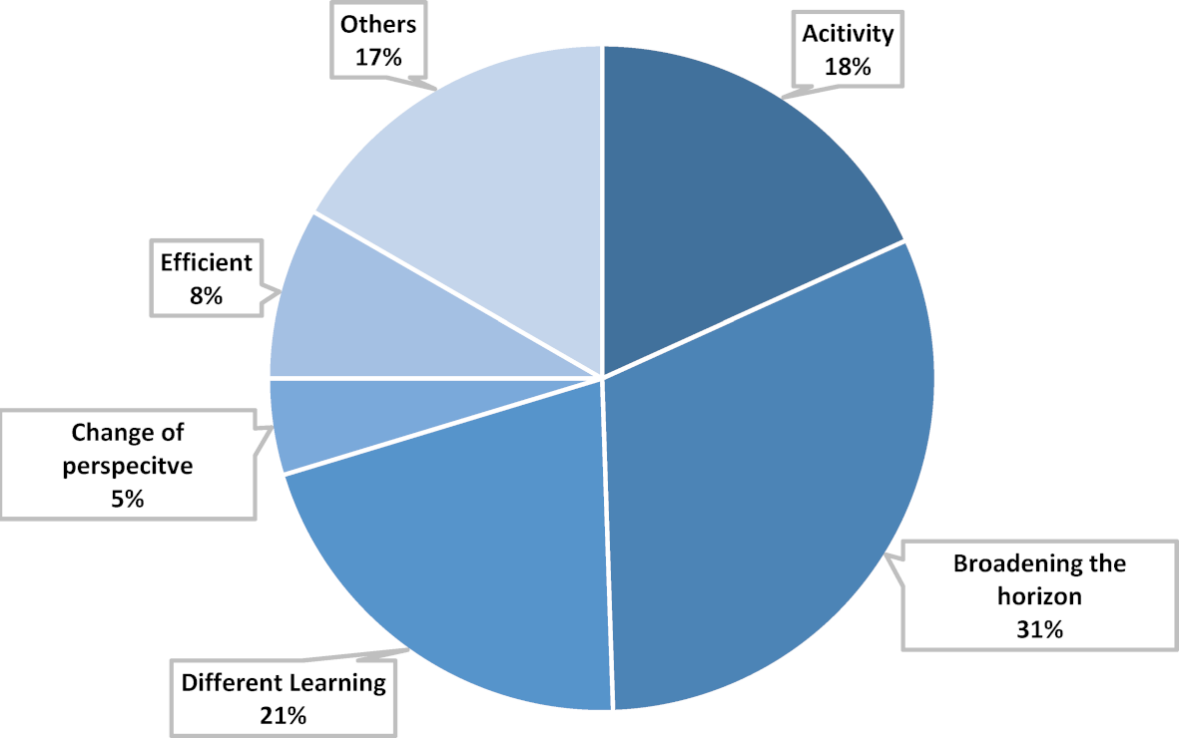
Main category Learning Success, n=192

The learning success is assessed by the participants as longterm (see figure 6 (Fig. 6)). In comparison with other courses in medical studies, the learning success here is rated greater and more sustainable. Moreover, the learning success is particularly high in comparison to the time spent. Accordingly, the time was used very well. Furthermore, the event contributed to the increased interest in the topic and the students want to continue to deal with topics of digital medicine after the end of the course. The fact that the students talk about wanting to continue learning after the course and to obtain further information in their free time represents a learning success. Learning in this class was less about conveying facts, but more about experiences and lessons learned. It is a different way of learning compared to other courses. During the compulsory elective week, critical reflection was encouraged. In the statements of the participants it is clear that the students link their learning success with activities. Thus, the practical experience of different media, techniques and devices and the subsequent discussion about them has contributed to the fact that the learning success is rated as particularly high. When asked about their personal learning success, many participants mention the concept of broadening horizons. The scope of the topic has now become clear to them, their view has been broadened by different perspectives and they feel “enlightened” about the topic of digital transformation in medicine. The numerous impulses of the lessons encouraged them to think about the topic and sensitized the students to the subject.

#### Main category learning experience

**Figure 7:**
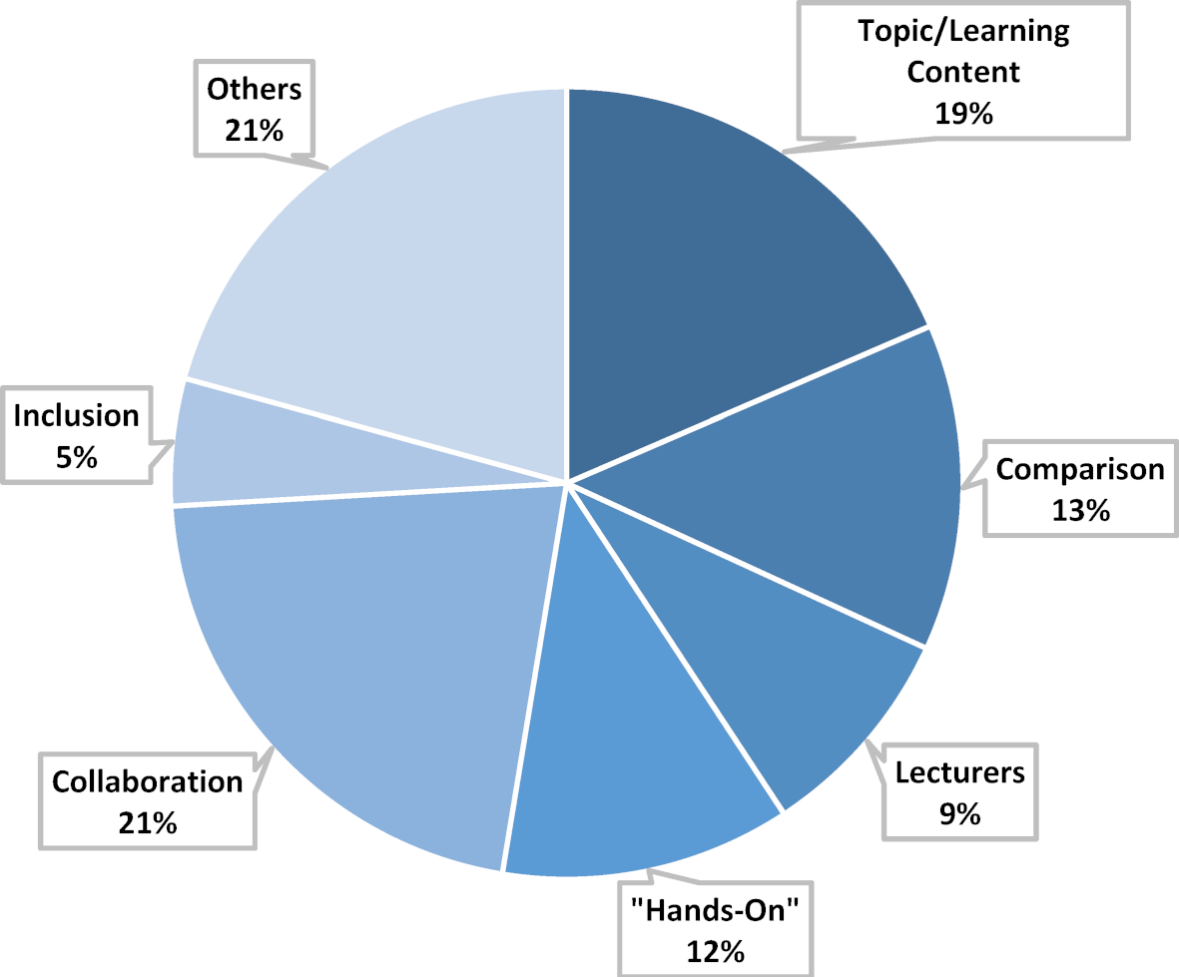
Main category Learning Experience, n=135

Participants describe their learning experiences in this event by referring to their learning experiences with different methods (see figure 7 (Fig. 7)). Statements on the topic of learning experience particularly often refer to the methods “collaboration”, “discussion” and “expert talk”. It is explained that the diversity of perspectives in discussions leads to a learning process, as does the opportunity to experiment with technology and media in small groups. Being able to work “hands on” on the DaVinci robot is described by many participants as an impressive learning experience. In addition, the students evaluate the variety of topics in the lessons positively. The fact that they were allowed to set their own learning goals is also emphasized. Overall, the respondents describe their learning experience during the event as extraordinarily varied compared to their other studies and feel it is sustainable. Not only the variety of topics, but also the topic itself is highlighted as an advantage of the course. The students state that they chose the course because of the topic that interests them. In addition, the learning content is described as exciting. Furthermore, the students emphasize the importance of the lecturer The fact that different co-lecturers were involved on a daily basis is mentioned as a positive aspect. It is emphasised that the lecturers met the students “at eye level” and were open to any questions.

#### Main category conclusion

**Figure 8:**
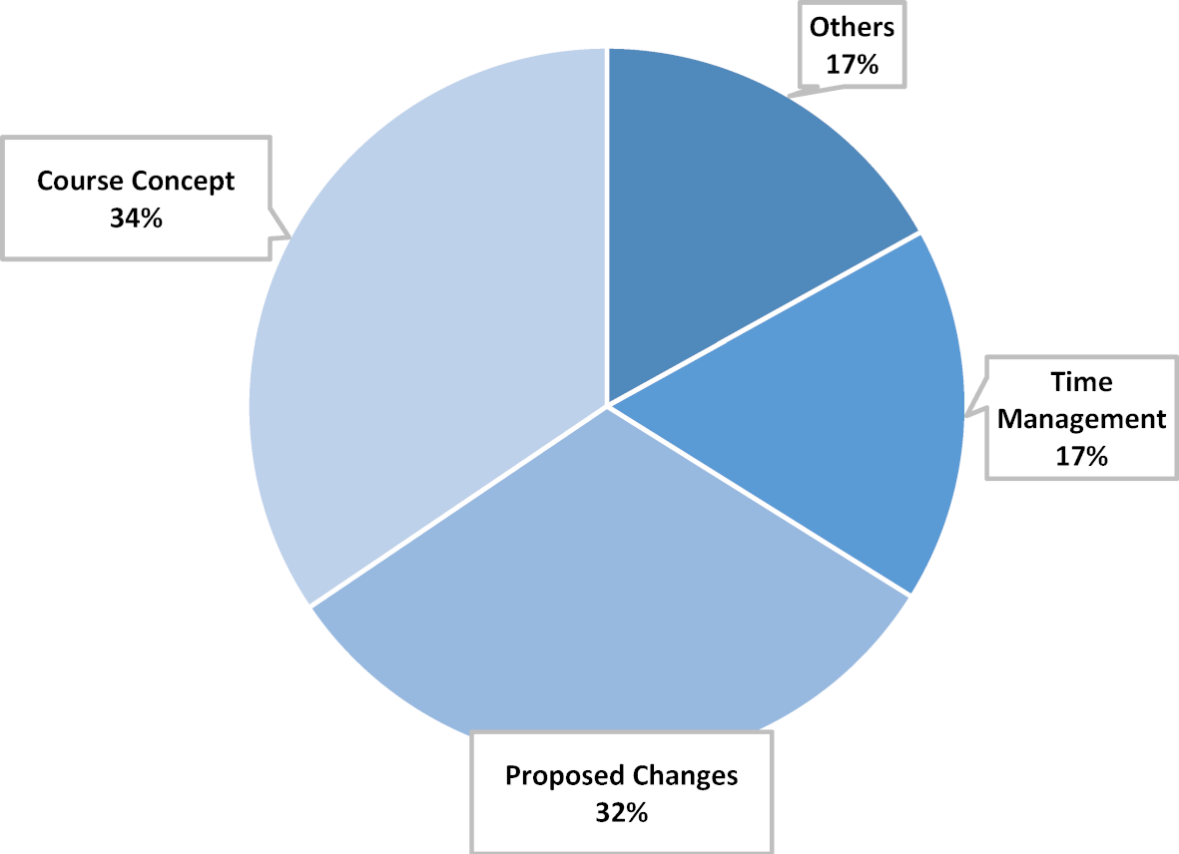
Main category Conclusion, n=177

Asked for a final evaluation of the event, almost all respondents expressed a positive opinion about the elective week (see figure 8 (Fig. 8)). The participants say that they learned a lot of new things and gained impressive experiences. Therefore, they recommend this course to others and hope that “Medicine in the digital age” will become part of the compulsory curriculum of medical studies. Although the organisation of the event is praised overall, criticism of time management is also expressed. The participants would like more time for discussions, expert talks and practical exercises and would accept longer classroom sessions for this. In addition, more breaks should be planned. Some participants would also like to see the proportion of practical exercises increased even further and to be able to try out even more “hands on”. For example, they would like to be able to test the software themselves in radiology, or to immerse themselves in the subject of app design.

In comparison to their other medical studies, “Medicine in the digital age” is not about accumulating as much factual knowledge as possible, but about a deeper understanding of the topics and the development of their own opinion. In addition, the motivation of the lecturers is noticeably high, which contributes to an exciting event.

## Discussion

The evaluation method is particularly suitable for course concepts that emphasize problem-centred learning, since the interviews not only allow for classical evaluation questions, but also for (critical) exchange, positioning and placing of learning in the individual experience context. Furthermore, the cooperation, which serves as a central didactic element in the context of “Medicine in the digital age”, is transferred to the evaluation phase, since the students' feedback is incorporated into the further development of the course. The method finds its limits in the intersubjective comparability and the objective measurement of learning progress. Furthermore, it should be noted that the students consciously decide to participate during the elective week, so that it can be assumed that at least a certain interest in the topic is already present. 

The results of the evaluation show that the course concept “Medicine in the digital age” is widely accepted. The students regret that the concept can only be taken as part of the compulsory elective week and express the urgent need for longitudinal implementation. The participants would like more time for discussions, expert talks and practical exercises and would accept longer classroom sessions for this. The wish was expressed that the proportion of practical exercises be increased even further. This feedback was taken up in the context of the iterative development. These include modifications in content (e.g. intensification of the AI topic), methods (additional hands-on workshops) and the composition of the lecturers (e.g. greater inclusion of patients as co-lecturers). The methods used are perceived by the students as appropriate and conducive to learning. The results provide important starting points for curriculum development for the digital age, which must not only promote technological application skills in the field of digital medicine, but must also include specific digital health applications, treatment concepts and decision support systems [3], [11], [16]. In addition to these technological aspects, basic digital literacy skills which address the reflection of social, ethical and legal contexts should be taught in particular. This reflection of attitudes is not bound to specific technical solutions but can be transferred to the overall context of digital transformation. In addition, classical key competencies need to be promoted, since skills such as creativity, self-reflection, problem-solving strategies and critical thinking will become increasingly important, especially in the context of an growing interdisciplinary, constantly changing medical landscape [17]. 

Current processes within the medical faculties and IMPP-associated reform efforts have recognized this need. Proportions of the concept and the results of the evaluation could give impulses to some medical faculties (including UKE Hamburg, Charité Berlin) in the design of similar teaching programs. In addition, digital competencies are a central cross-sectional topic in the joint reform efforts of the competency-based learning objectives catalogue of the medical faculties and the state board exam. The main author was able to contribute both the learning objectives and the results of the evaluation. Furthermore, extensive parts of the evaluation were included into the development processes of the German Medical Association’s continuing medical education curriculum “Digital Health Applications in Clinics and Practices” as well as several central national strategy processes of the University Forum on Digitization. These include the main topics and publications on “Curriculum Development in the Digital Age" and "Digital Literacy” and “Data Literacy” [10], [11], [16], [17]. 

The development of a digitisation strategy and its didactic is therefore a relevant component of future planning for the curricular development of medical school, but also for continuous medical education. In the future, this will no longer be possible with a compulsory elective course but requires a comprehensive implementation in the curriculum. In this context, it must be critically reflected whether and how the course can be scaled. We are convinced that the practical and reflective parts must be represented in the form of internships for a maximum of 15 students. When developing these curricula, the high speed of the change process should also be taken into account and curricular adaptation in the sense of “agility by design” should be made possible right from the conception stage [10].

## Funding

The project “Medicine in the digital age” was funded by the Stifterverband as part of the support program Curriculum 4.0 and by the Reinhard Frank Foundation as part of “Digital medical studies – artificial intelligence and diagnosis” and “Doctor 2022”. 

## Competing interests

The authors declare that they have no competing interests. 

## Supplementary Material

Interview guide medicine in the digital age
