# Supplementary material for: Digital skills for medical students – qualitative evaluation of the curriculum 4.0 “Medicine in the digital age”
Source: GMS J Med Educ. 2020 Nov 16;37(6):Doc60. doi: 10.3205/zma001353 (PMC7672383; doi:10.3205/zma001353)
Supplement: Interview guide medicine in the digital age [file JME-37-60-s-001.pdf]

Attachment 1: Interview guide medicine in the digital age

- What does the advancing technical development mean for your role as a doctor?
- What does the advancing technical development mean for the patient?
- Where do you see challenges and chances of this development?
- How do you position yourself/yourself for the use of technical innovations (AI, robotics, telemedicine) in medical practice?
- How has your interest in the digital transformation of medicine changed through your participation?
- Do you have (ethical/moral) concerns?
- How do you/they judge the amount of time required in terms of learning success.
- What did you like/have in the teaching concept and what did you miss?
- How does the learning success in "Medicine in the digital age" differ from the learning success due to your other learning habits?
- How do you rate your personal learning experience?
- What is your overall conclusion of the course "Medicine in the digital age"?
- Is there anything else you want to say?

Attachment 1 to: Kuhn S, Müller N, Kirchgässner E, Ulzheimer L, Deutsch KL. *Digital skills for medical students – qualitative evaluation of the curriculum 4.0 “Medicine in the digital age”*. GMS J Med Educ. 2020;37(6):Doc60. DOI: 10.3205/zma001353
